# Detection and characterization of multidrug-resistant *Klebsiella* spp. and *Enterobacter* spp. isolates in Colombian guinea pigs intended for human consumption

**DOI:** 10.14202/vetworld.2025.3187-3196

**Published:** 2025-10-31

**Authors:** Tatiana Gabriela Paz-Calvache, Sofía J. Merchancano, Luciano Nakazato, Valeria Dutra

**Affiliations:** 1 Department of Microbiology and Molecular Biology, College of Veterinary Medicine, Faculty of Veterinary Medicine, Federal University of Mato Grosso, Cuiabá, Mato Grosso, Brazil; 2 Veterinary Medicine Program, Faculty of Animal Science, University of Nariño. Pasto, Colombia

**Keywords:** antimicrobial resistance, *Cavia porcellus*, *Enterobacter*, guinea pig, *Klebsiella*, multidrug resistance, resistance genes

## Abstract

**Background and Aim:**

Antimicrobial resistance (AMR) has emerged as a global health threat, with food-producing animals recognized as reservoirs of multidrug-resistant (MDR) bacteria. Guinea pigs (*Cavia porcellus*), traditionally consumed in several Andean regions, remain underexplored in terms of food safety risks. This study aimed to detect, characterize, and analyze the AMR patterns and resistance genes of *Klebsiella* spp. and *Enterobacter* spp. isolated from slaughtered guinea pigs intended for human consumption in Colombia.

**Materials and Methods:**

A cross-sectional descriptive study was conducted on 70 guinea pigs with macroscopic intestinal lesions. Intestinal swabs were cultured on blood and MacConkey agar, and isolates were identified using biochemical tests and 16S ribosomal RNA sequencing. Antimicrobial susceptibility testing was performed against nine antibiotics representing seven antimicrobial classes, using Clinical and Laboratory Standards Institute standards. MDR was defined as resistance to ≥1 antibiotic in ≥3 classes. Polymerase chain reaction assays were employed to detect resistance genes, including β-lactamase *Klebsiella pneumoniae* carbapenemase (*bla_KPC_*), class d β-lactamase oxacillinase-48 gene (*bla_OXA-48_*), ampC-lactamase (*ampC*), and New Delhi metallo-beta-lactamase 1 (*bla_NDM-1_*).

**Results:**

Ten isolates were obtained, comprising *Klebsiella* spp. (70%) and *Enterobacter hormaechei* (30%). All isolates (100%) exhibited MDR profiles. High resistance rates were observed against fluoroquinolones (100%), beta-lactams (90%), aminoglycosides (70%), carbapenems (70%), and cephalosporins (70%), whereas resistance to trimethoprim–sulfamethoxazole was 40%. Molecular analysis revealed the presence of *bla_KPC_* in seven isolates, *bla_OXA-48_* in two, and *ampC* in three. No *bla_NDM-1_* genes were detected. Notably, *E. hormaechei* isolates demonstrated broader resistance spectra than *Klebsiella*.

**Conclusion:**

This study provides the first evidence in Colombia of MDR *Klebsiella* spp. and *Enterobacter* spp. isolates carrying clinically important resistance genes in guinea pigs intended for human consumption. The detection of carbapenemase genes (*bla_KPC_* and *bla_OXA-48_*) is particularly concerning given their role in limiting therapeutic options. These findings highlight the urgent need for improved antimicrobial stewardship, stricter regulation of antibiotic use in guinea pig production systems, enhanced surveillance, and targeted farmer education to mitigate AMR risks at the human–animal interface.

## INTRODUCTION

The rising morbidity and mortality linked to antimicrobial resistance (AMR) have become a pressing global concern, exacerbated by the emergence and re-emergence of pathogenic species, zoonotic transmission, and treatment-resistant infections. Domestic animals serve as important reservoirs and vectors for resistant bacteria, including *Enterococcus* spp., *Escherichia coli*, *Klebsiella pneumoniae*, methicillin-resistant *Staphylococcus aureus*, vancomycin-resistant *Enterococcus*, *Acinetobacter baumannii*, *Pseudomonas aeruginosa*, and *Enterobacter* spp. [1].

The indiscriminate use and misuse of antibiotics have fueled the rise of multidrug-resistant (MDR) strains, posing a major threat to global health. Notably, *K. pneumoniae* and *Enterobacter* spp. are among the *Enterococcus* faecium, *Staphylococcus aureus*, *Klebsiella pneumoniae*, *Acinetobacter baumannii*, *Pseudomonas aeruginosa*, and *Enterobacter* spp. (ESKAPE pathogens), a group of bacteria notorious for causing severe infections that are increasingly difficult to treat [2]. These microorganisms are ubiquitous in the environment and can act as opportunistic pathogens in plants, animals, and humans [3]. In Colombia, the guinea pig production chain is particularly vulnerable due to limited technological support and inadequate biosecurity measures. When coupled with the poor sanitary conditions commonly observed in smallholder farms and townships, these factors create an enabling environment for the spread of parasitic, viral, and bacterial diseases, including those driven by MDR pathogens [4, 5].

Despite extensive research on AMR in major livestock species such as poultry, swine, and cattle, there is a striking paucity of information on AMR in small animals raised for human consumption, particularly guinea pigs *(Cavia porcellus*). Guinea pig farming holds cultural and nutritional significance across Andean countries, including Colombia, yet studies addressing bacterial pathogens and their resistance mechanisms in this production system remain scarce. Previous investigations have primarily focused on common foodborne pathogens in conventional livestock, while data on the prevalence, resistance profiles, and genetic determinants of *Klebsiella* spp. and *Enterobacter* spp. in guinea pigs are virtually absent. This knowledge gap is particularly concerning because these bacteria are members of the ESKAPE group of pathogens, which are responsible for difficult-to-treat infections in humans and are increasingly associated with the spread of resistance genes across the food chain. Furthermore, suboptimal sanitary practices and the widespread, often unregulated, the use of antibiotics in guinea pig production in Colombia may create conditions conducive to the emergence and dissemination of MDR strains. However, no systematic studies have yet examined the occurrence of resistance genes such as *bla_KPC_*, *bla_OXA-48_*, or *ampC* in bacteria isolated from guinea pigs intended for human consumption.

The present study aimed to address this knowledge gap by detecting and characterizing MDR *Klebsiella* spp. and *Enterobacter* spp. isolated from slaughtered guinea pigs with intestinal lesions in Colombia. Specifically, the objectives were (i) to determine the prevalence of these bacterial species in guinea pig carcasses, (ii) to assess their AMR patterns across multiple drug classes, and (iii) to identify key resistance genes associated with clinically important resistance mechanisms. By integrating phenotypic and molecular approaches, this study provides essential baseline data for understanding the potential public health risks posed by guinea pig meat as a reservoir of AMR bacteria. The findings aim to inform future surveillance, promote antimicrobial stewardship, and support the development of biosecurity and regulatory strategies to mitigate the dissemination of resistance through non-traditional food animal systems.

## MATERIALS AND METHODS

### Ethical approval

This study was conducted in accordance with ethical guidelines and was approved by the Bioethics Committee of the University of Nariño, Colombia (Approval No. 079-2023).

### Study period and location

The study was conducted from January to June 2023. Samples were collected from guinea pigs sent for slaughter in the municipality of Pasto (1°12’23.7”N 77°16’55.9”W) and its administrative units, such as Cabrera (1°12’55.239”N 77°12’52.080”W) and Catambuco (1°10’0.714”N 77°17’42.125”W). In addition, these slaughter sites receive guinea pigs from family and commercial farms in the municipality of Pasto and from other municipalities in the Nariño region, such as Ipiales (0°49’47.2”N 77°38’30.9”W) and Tambo (1°24’18.978”N 77°23’20.323”W).

### Study design and sampling

A cross-sectional descriptive study was performed on 70 guinea pig carcasses (32 males and 38 females) obtained from slaughterhouses in Pasto, Nariño, Colombia. Only animals presenting macroscopic intestinal lesions during slaughter were included. Intestinal swabs were collected using Probact Cary Blair transport medium (TS/5-21; Cary Blair, UK).

### Inclusion criteria

Animals of both sexes, weighing >1 kg, aged >2 months, and showing macroscopic intestinal lesions characterized by intense red to dark reddish-black coloration.

### Exclusion criteria

Animals without intestinal lesions were excluded from the study.

### Bacterial isolation and identification

All 70 intestinal swab samples were plated on 8% sheep blood agar (Lot No. M3GOKX01, TM Media, Delhi) and MacConkey agar (Lot No. 601101, KASVI, Curitiba). Plates were incubated at 36°C for up to 48 h. Colonies obtained were mucoid, white to gray, round, flat, and large; all isolates were Gram-negative rods.

Biochemical testing revealed a yellow/yellow triple sugar iron reaction with gas production, citrate positivity, indole negativity, lactose fermentation, catalase positivity, and oxidase negativity. *Klebsiella* isolates were non-motile, while *Enterobacter hormaechei* isolates exhibited motility. These findings are consistent with previously reported findings [6] and are presented in Supplementary Table.

### Antimicrobial susceptibility testing

Antimicrobial susceptibility was assessed by disk diffusion using nine antibiotics (Cecon, São Paulo, Brazil) representing seven classes:

- Amoxicillin (AMO, 10 μg; batch 9079)
- Ampicillin (AMP, 10 μg; batch 9241)
- Ceftiofur (CTF, 30 μg; batch 7821)
- Ciprofloxacin (CIP, 5 μg; batch 8137)
- Doxycycline (DOX, 30 μg; batch 8723)
- Enrofloxacin (ENO, 5 μg; batch 7653)
- Gentamicin (GEN, 10 μg; batch 9532)
- Meropenem (MPM, 10 μg; batch 7769)
- Sulfamethoxazole–trimethoprim (SU-TRI, 25 μg; batch 9200).

*E. coli* ATCC 25922 was used as the quality control strain. Zone diameters were interpreted following Clinical and Laboratory Standards Institute (CLSI, 2024; M100 performance standards). As CLSI breakpoints for guinea pigs are not established, resistance was interpreted using standards for humans and other animal species. Isolates were classified as MDR when resistant to ≥1 antibiotic in ≥3 antimicrobial classes, in accordance with international consensus guidelines [2].

### DNA extraction and molecular identification

Genomic DNA was extracted using the MagMAX CORE Nucleic Acid Purification Kit (Applied Biosystems, Cat. No. A32700; Thermo Fisher Scientific, Austin, TX, USA). DNA was eluted in 70 μL ultrapure water and stored at ACTGene, RS, Brazil. Concentration and purity were measured spectrophotometrically at 260/280 nm, and integrity was verified [3].

Polymerase chain reaction (PCR) amplification of the 16S ribosomal RNA (rRNA) gene was conducted [7], and sequences were compared with GenBank data using the National Center for Biotechnology Information (NCBI) Basic Local Alignment Search Tool (BLAST) [8]. Positive controls included cloned PCR amplicons in the pGEM-T plasmid (Promega) transformed into *E. coli* DH5-α cells (*DH5-α*) a laboratory cloning strain of *E. coli* commonly used for plasmid propagation, verified by Sanger sequencing. Internal PCR controls used previously tested 16S rRNA-positive samples, whereas negative controls contained ultrapure water. Detailed PCR conditions are presented in Supplementary Table.

### Detection of resistance genes

PCR assays targeted resistance genes (*bla_OXA-48_*, *bla_NDM-1_*, *ampC*, and *bla_KPC_*). PCR products were resolved by agarose gel electrophoresis at 6 V/cm, stained with GelRed nucleic acid stain (Biotium, Fremont, CA, USA), and visualized under UV light. Products were purified and sequenced using a genetic analyzer (Applied Biosystems, USA [ABI 3500]), automated Sanger sequencer, and sequences were analyzed with the NCBI BLAST tool [8]. Sequence editing and alignments were performed using CLC DNA Workbench 6.0 (Qiagen, Hilden, Germany). Detailed amplification conditions are provided in Supplementary Table.

## RESULTS

### Bacterial identification

Out of 70 guinea pig carcasses examined, ten bacterial isolates were recovered. Macroscopic, morphological, staining, and biochemical characteristics identified 70% of the isolates as *Klebsiella* spp. and 30% as *Enterobacter* spp. [8]. These findings were further confirmed through 16S rRNA sequence amplification and analysis. The detailed species-level identification is presented in Table 1.

**Table 1:** Bacteria isolated from intestinal swabs of *Cavia porcellus*.

Bacteria	Frequency in percentage
*Klebsiella grimontii*	20
*Klebsiella pasteurii*	30
*Klebsiella aerogenes*	20
*Enterobacter hormaechei*	30

### Overall AMR profiles

Antimicrobial susceptibility testing demonstrated that all isolates (100%) exhibited MDR. The highest resistance rates were observed against beta-lactams and fluoroquinolones (90%–100%), followed by aminoglycosides (70%), carbapenems (70%), and cephalosporins (70%). Resistance to diaminopyrimidines was comparatively lower at 40%. Detailed AMR patterns are summarized in Table 2.

**Table 2:** Bacteria isolated from guinea pigs and their resistance patterns based on human CLSI and veterinary CLSI values.

Bacteria	*K. grimontii*	*K. grimontii*	*K. pasteurii*	*K. pasteurii*	*K. pasteurii*	*K. aerogenes*	*K. aerogenes*	*E. hormaechei*	*E. hormaechei*	*E. hormaechei*
AMP										
Value mm	0	9	10	10	9	15	12	0	0	15
CLSI Humans (S ≥ 17, R ≤ 13)	**R**	**R**	**R**	**R**	**R**	**R**	**R**	**R**	**R**	**R**
CIP										
Value mm	24	22	21	24	17	22	19	18	10	23
CLSI Human (S ≥ 26, R ≤ 21)	**R**	**R**	**R**	**R**	**R**	**R**	**R**	**R**	**R**	**R**
ENO										
Value mm	22	18	8	23	16	20	18	18	9	23
CLSI Cats (S ≥ 23, R < 16)	**R**	**R**	**R**	S	**R**	**R**	**R**	**R**	**R**	S
CLSI Poultry (S ≥ 23, R < 16)	**R**	**R**	**R**	S	**R**	**R**	**R**	**R**	**R**	S
AMO										
Value mm	21	7	11	10	11	14	15	0	0	9
CLSI Humans (S ≥ 17, R ≤ 13)	S	**R**	**R**	**R**	**R**	**R**	**R**	**R**	**R**	**R**
DOX										
Value mm	14	17	0	15	13	10	10	14	10	14
CLSI Humans (S ≥ 14, R ≤ 10)	S	S	**R**	S	**R**	**R**	**R**	S	**R**	S
GEN										
Value mm	12	16	16	27	15	20	18	11	11	15
CLSI Humans (S ≥ 18, R ≤ 14)	**R**	**R**	**R**	S	**R**	S	S	**R**	**R**	**R**
CLSI Dogs (S ≥ 16, R ≤ 12)	**R**	S	S	S	**R**	S	S	**R**	**R**	**R**
CLSI Horses (S ≥ 16, R ≤ 12)	**R**	S	S	S	**R**	S	S	**R**	**R**	**R**
CTF										
Value mm	18	22	18	23	16	15	15	20	16	22
CLSI Pigs (S ≥ 21, R ≤ 17)	**R**	S	**R**	S	**R**	**R**	**R**	**R**	**R**	S
MPM										
Value mm	25	25	21	26	20	21	15	20	12	18
CLSI Humans (S ≥ 23, R ≤ 19)	S	S	**R**	S	**R**	**R**	**R**	**R**	**R**	**R**
SU_TRI										
Value mm	24	20	12	14	16	23	17	14	9	16
CLSI Humans (S ≥ 16, R ≤ 10)	S	S	**R**	**R**	S	S	S	**R**	**R**	S

**R** = Resistant isolates based on veterinary and human CLSI values, S = Sensible isolates based on veterinary and human CLSI values, CLSI = Clinical and Laboratory Standards Institute, *K. grimontii = Klebsiella grimontii, K. pasteurii = Klebsiella pasteurii, K. aerogenes = Klebsiella aerogenes, E. hormaechei = Enterobacter hormaechei,* AMO = Amoxicillin, AMP = Ampicillin, CTF = Ceftiofur, CIP = Ciprofloxacin, DOX = Doxycycline, ENO = Enrofloxacin, GEN = Gentamicin, MPM = Meropenem, SU-TRI = Sulfamethoxazole–trimethoprim.

### Comparative resistance between *Klebsiella* and *Enterobacter*

*Enterobacter* isolates showed higher proportional resistance despite their lower frequency. They exhibited 100% resistance to nearly all tested antibiotics, with partial susceptibility to CTF, ENO, SU-TRI (33.3% susceptible), and DOX (66.7% susceptible). In contrast, *Klebsiella* isolates displayed a broader distribution of resistance, ranging from 28.5% to 100%, with higher absolute numbers of resistant strains.

### Resistance by antimicrobial class

- Beta-lactams: The highest resistance rates were observed among *K. pasteurii*, *K. aerogenes*, and *E. hormaechei*. *K. grimontii* displayed the lowest beta-lactam resistance.
- Fluoroquinolones: All isolates (100%) were resistant. CIP resistance was observed in 20% of *K. aerogenes* and *K. grimontii*, 30% of *K. pasteurii*, and 30% of *E. hormaechei*. According to feline and poultry CLSI standards, 80% of isolates were resistant to ENO (60% *Klebsiella* spp., 20% *E. hormaechei*).
- Tetracyclines: DOX resistance was found in 50% of isolates, including 40% *Klebsiella* spp. and 10% *E. hormaechei*.
- Aminoglycosides: GEN resistance was observed in 70% of isolates, including 30% *E. hormaechei* and 20% of both *K. grimontii* and *K. pasteurii*.
- Carbapenems: MPM resistance was present in 40% of *Klebsiella* spp. (20% *K. aerogenes* and 20% *K. pasteurii*) and 30% of *E. hormaechei*.
- SU-TRI: The lowest resistance rate was recorded at 40%, including 20% *E. hormaechei* and 20% *K. pasteurii*.
- Cephalosporins: Based on swine CLSI standards, 70% of isolates were resistant to CTF, including three *Klebsiella* spp. and two *E. hormaechei* isolates.

### Resistance genes

Molecular screening showed no detection of the *bla_NDM-1_* gene. However, key resistance determinants were identified:

- *bla_KPC_* detected in five *Klebsiella* spp. and two *E. hormaechei* isolates.
- *ampC* gene present in two *Klebsiella* spp. and one *E. hormaechei* isolate.
- *bla_OXA-48_* identified in two *E. hormaechei* isolates.

## DISCUSSION

This study, which is one of the few conducted on guinea pigs and the first of its kind in Colombia and Brazil, revealed a high prevalence of AMR in potentially zoonotic bacteria. These findings are consistent with those of a previous study by Vázquez *et al*. [9], which reported *Klebsiella* isolation (11.7%) in fecal samples from guinea pigs raised in family and commercial systems in a different region.

### Antibiotic use and stewardship in Latin America

As the global demand for animal protein increases, the judicious use of antibiotics, whether as growth promoters or for preventive and prophylactic purposes, remains a challenge. In recent years in Latin America, hospital antimicrobial stewardship program (AMS) initiatives have been implemented mostly in the capital cities of Brazil, Argentina, Colombia, Cuba, Mexico, and Chile. However, control policies are still quite flexible in Colombia, which is why the purchase and sale of antibiotics without a medical prescription is a factor that has an impact on AMR [10]. In this context, analyzing the consequences of this practice for the emergence of resistant strains and exploring effective strategies to mitigate this growing problem in food products, where information is scarce, is important, as seen in the consumption of guinea pigs.

### Pathogenic potential of *Enterobacter* and *Klebsiella*

*Enterobacter* includes several highly resistant bacterial pathogens and is a major contributor to nosocomial infections. These bacteria inhabit diverse environments, including soil, water, and the intestinal microbiota of humans and animals, and are also phytopathogenic to several plant species [11]. *Klebsiella* is a Gram-negative bacterium commonly found in natural environments and in the human microbiota. *Klebsiella* infection is also associated with the onset and progression of various diseases. Consequently, research has focused on identifying effective antimicrobials and elucidating MDR mechanisms, especially against carbapenems and beta-lactams [12].

### Resistance in *K. aerogenes* and clinical implications

*K. aerogenes* isolates showed resistance to five antimicrobials based on human CLSI standards and two based on veterinary standards. This is significant because *K. aerogenes* commonly causes infections in immunocompromised patients in hospital settings, leading to urinary tract infections and pneumonia with elevated morbidity and mortality [13]. The high level of resistance complicates the treatment of nosocomial infections, as available antimicrobial options become less effective, hindering clinical management. In addition, their dual role as commensals and opportunistic pathogens underscores their importance in both health and disease [13].

### Foodborne transmission and public health risks

Various *Klebsiella* strains are major contributors to nosocomial infections in humans. Strains isolated from broiler chicken meat have also been found [14], suggesting that contaminated meat may serve as a transmission route, potentially causing disease in consumers. This emphasizes the importance of monitoring bacterial prevalence and resistance patterns in food products.

### Antibiotic misuse in guinea pig farming

Widespread MDR in foodborne bacteria is largely due to inappropriate antimicrobial usage, particularly beta-lactams, in livestock production [14]. In guinea pig farming, ENO, SU-TRI, and oxytetracycline are the most frequently used antimicrobials prescribed by untrained personnel (88%) [15]. This study reported a high resistance rate to fluoroquinolones because isolates with intermediate or resistant phenotypes to one fluoroquinolone are more likely to develop cross-resistance to the entire class (CLSI, 2024; M100 performance standards). Intestinal isolates from guinea pigs showed the highest resistance to beta-lactams (AMP and AMO), followed by GEN, fluoroquinolones (70%–100%), and SU-TRI (40%). Workers involved in slaughtering, evisceration, and carcass cleaning often fail to use proper biosafety equipment, increasing their exposure to antimicrobial-resistant pathogens.

### Comparisons with other studies

A study by Wu *et al*. [14] on *Klebsiella* strains isolated from chicken meat reported higher resistance rates to cephalosporins and beta-lactams (100% and 98.9%, respectively), with slightly lower resistance to CIP (80%). Our findings of 100% resistance to CIP and beta-lactams are consistent with those reported in the aforementioned study. However, CIP resistance in the study by Aleshina *et al*. [16] was significantly lower (27%) than that in our study. In Colombia, first-generation cephalosporins are the most commonly used antimicrobials within the beta-lactam group (J01D), accounting for approximately 70% of total consumption. They are followed by third-generation cephalosporins, with 9.4%, whereas second- and fourth-generation cephalosporins represent 5% and 5.7% of total consumption, respectively [17]. This pattern of antibiotic use may contribute to the high levels of antibiotic resistance observed in these bacteria.

### Resistance to ENO and carbapenems

This study demonstrated ENO resistance rates as high as 80%. These results contrast with a study by Aleshina *et al*. [16], which reported lower ENO resistance rates of approximately 25% in *Klebsiella* spp. Nevertheless, this is a noteworthy observation, given the widespread use of ENO in guinea pig production systems [15].

According to data reported by the Global Antimicrobial Resistance and Use Surveillance System for 2022, carbapenem use in Colombia is considered moderate, accounting for 9.4% of total beta-lactam consumption. This is concerning, given that carbapenems are reserved for the treatment of severe infections caused by MDR bacteria [17]. Moreover, carbapenem resistance is a critical concern in *Klebsiella* spp., with reported resistance rates ranging from 96% to 100% [18, 19]. Typically, it shows lower rates of carbapenem resistance, ranging from 18% to 25% [16]. MPM resistance in guinea pig isolates appeared to be lower (70%) when interpreted using human CLSI breakpoints. This is particularly relevant because carbapenemase-producing *Klebsiella* spp. are increasingly common globally and pose serious public health risks. MPM remains a broad-spectrum agent that is effective against MDR strains and is stable in the presence of beta-lactamases, including extended-spectrum beta-lactamases [20].

### GEN resistance and usage concerns

GEN exhibited the highest resistance rate, affecting 70% and 50% of the isolates under both human and veterinary CLSI standards, respectively. This finding is important because antibiotic sales in Colombia are poorly regulated, and farmers often fail to adhere to medication withdrawal periods [15]. Therefore, the use of GEN in food-producing animals should be limited due to its long withdrawal period and high residue risk [21].

### Intrinsic and comparative resistance patterns

Although *Klebsiella* spp. and *Enterobacter* spp. are intrinsically resistant to AMP and AMO, these antibiotics were included in the susceptibility testing, following the CLSI standards, to allow comparison with previous studies in both animals and humans. The high resistance observed in this study was consistent with earlier reports and reflected the resistance characteristics of *Klebsiella* spp. and *Enterobacter* spp. [22–26]. In contrast, 40% of isolates were resistant to SU-TRI, a rate equal to that reported in a previous Ecuadorian study by Vázquez *et al*. [9], which found 40% resistance (11/28 *Klebsiella* isolates) in animals with gastrointestinal symptoms.

### Significance of *E. hormaechei* resistance

The resistance observed in *E. hormaechei* isolates is also significant because this species is an opportunistic pathogen capable of infecting both humans and animals. Several reports have indicated that resistant bacteria are shed into the environment through feces. The detection of *E. hormaechei* in chicken feed suggests the potential transmission of resistance genes to the intestinal microbiota through exposure to feed. This may be related to the current findings, as isolates were recovered from the intestinal tract of *C. porcellus* [27].

### Distribution of resistance genes

*bla_KPC_* was more prevalent in *Klebsiella* spp. than in *Enterobacter* spp. In our study, two of three *E. hormaechei* isolates and five of seven *Klebsiella* isolates carried this gene, despite limited data on *bla_KPC_* production in *Enterobacter* spp. [28]. The *Klebsiella* carriers included *K. grimontii* (one isolate), *K. aerogenes* (two isolates), and *K. pasteurii* (two isolates), although *bla_KPC_* is more commonly found in *K. pneumoniae* and is relatively rare in other species [27]. While a study by Ceylan *et al*. [29] reported a low prevalence of *bla_KPC_* (17.9%), while Ferreira *et al*. [30] found it in all isolates. Similar findings were observed in guinea pigs, suggesting that the presence of this gene could be geographically related to the isolates’ location [31].

In this study, carbapenem-resistant isolates showed a low prevalence of the *bla_OXA-48_* gene, with only 20% of isolates carrying it. The *bla_OXA-48_* gene is commonly associated with carbapenem resistance in *Enterobacter*iaceae, and its presence in this family is often linked to high antibiotic resistance [29]. In our analysis, only 28.57% of the carbapenem-resistant isolates (70%) harbored *bla_OXA-48_*, which contrasts with previous studies reporting that 97.7% of carbapenem-resistant *K. pneumoniae* strains carried this gene. Another study by Dedeić-Ljubović *et al*. [31] on hospitalized patients found that, among 71 isolates, 79.8% possessed *bla_OXA-48_*, whereas other resistance genes, such as *bla_NDM-1_ (*21.4%) and *bla_KPC_* (17.9%), were less prevalent. Although *bla_OXA-48_* is an important contributor to carbapenem resistance, its prevalence may vary across studies and bacterial populations.

### Study limitations

This study has some limitations. Advanced genetic analyses, such as multilocus sequence typing (MLST) or whole-genome sequencing (WGS), could not be performed on the bacterial strains due to resource limitations and the small number of isolates (n = 10), which restricted the ability to analyze strain diversity and their relatedness. As the focus was solely on animals that already exhibited signs of intestinal lesions, environmental, occupational, and slaughterhouse samples were not included; therefore, the findings do not provide a broader context regarding the bacteria’s origin or prevalence in the wider environment. Finally, the detection of virulence genes, such as regulator of regulator of the mucoid phenotype A (*rmpA*) or ferric aerobactin receptor (*iutA*), was not conducted because the scope of the project was limited to AMR genes.

## CONCLUSION

This study, the first of its kind in Colombia and Brazil, focusing on guinea pigs (*C. porcellus*), revealed a high prevalence of MDR *Klebsiella* spp. (70%) and *E. hormaechei* (30%) isolates from slaughtered animals with intestinal lesions. Antimicrobial susceptibility testing showed universal MDR profiles, with alarming resistance to fluoroquinolones (100%), beta-lactams (90%–100%), aminoglycosides (70%), carbapenems (70%), and cephalosporins (70%). Resistance genes of major clinical relevance, including *bla_KPC_*, *bla_OXA-48_*, and *ampC*, were detected, whereas *bla_NDM-1_* was absent. Notably, *E. hormaechei* isolates exhibited higher proportional resistance compared to *Klebsiella*, underscoring their potential as emerging reservoirs of AMR.

These findings underscore the significant food safety and public health risks associated with consuming guinea pig meat, a culturally significant protein source in the Andean region. The presence of carbapenemase-producing isolates (*bla_KPC_* and *bla_OXA-48_*) in food animals raises concern about possible transmission of resistance determinants to humans through direct contact, environmental contamination, or the food chain. The evidence calls for stricter regulation of antibiotic sales and use, implementation of AMSs in non-conventional livestock production, and improved farmer training on biosafety and responsible medication practices.

A major strength of this research lies in being the first systematic characterization of AMR in *Klebsiella* and *Enterobacter* from guinea pigs in Colombia. By combining phenotypic antimicrobial susceptibility testing with molecular detection of resistance genes, the study provides both epidemiological and genetic insights into MDR pathogens in this under-researched production system.

Further studies should employ advanced molecular tools such as MLST or WGS to unravel the clonal diversity and evolutionary dynamics of resistant strains. Broader surveillance, including healthy animals, environmental samples, and occupational settings, is needed to trace the origin and dissemination pathways of AMR. In addition, studies incorporating virulence factors would enhance understanding of the zoonotic potential of these isolates.

The detection of MDR *Klebsiella* and *Enterobacter* isolates carrying carbapenemase genes in guinea pigs underscores an urgent One Health concern. Strengthening antimicrobial stewardship, enforcing biosecurity measures, and enhancing AMR monitoring in non-conventional food animal systems are critical steps to mitigate the spread of resistance. Addressing these challenges is essential not only for safeguarding animal health but also for protecting human health and ensuring sustainable food production.

## DATA AVAILABILITY

The supplementary data can be made available from the corresponding author upon request.

## AUTHORS’ CONTRIBUTIONS

TGP and VD: Conceptualized and designed the study. TGP, VD, and LN: Conducted the experiment and data collection. TGP, VD, LN, and SJM: Interpretation of the results and drafted and critically revised the manuscript. All authors have read and approved the final version of the manuscript.
